# Entwicklung und Validierung eines Kurskonzepts zur medizinisch-taktischen Rettung unter Tage: Standardisiertes Ausbildungscurriculum für Grubenwehren

**DOI:** 10.1007/s00063-021-00861-w

**Published:** 2021-09-20

**Authors:** Frank Reuter, Andreas Fichtner, Benedikt Brunner, Denise Preuss, Beate Herrmann, Martin Herrmann

**Affiliations:** 1 Forschungs- und Lehrbergwerk, Technische Universität Bergakademie Freiberg, Freiberg, Deutschland; 2 Notfallaufnahme, Kreiskrankenhaus Freiberg gGmbH, Donatsring 20, 09599 Freiberg, Deutschland; 3 Scientific Diving Center, Lehrstuhl für Technische Thermodynamik, Technische Universität Bergakademie Freiberg, Freiberg, Deutschland; 4 Sächsisches Oberbergamt, Freiberg, Deutschland; 5 Forschungs‐ und Lehrbergwerk Reiche Zeche, Fuchsmühlenweg 9 a, 09599 Freiberg, Deutschland

**Keywords:** Notfallrettung, Bergbau, Kursentwicklung, Effektquantifizierung, Praktische Kompetenz, Emergency rescue, Mining, Course development, Effect quantification, Practical competency

## Abstract

**Hintergrund:**

Die Strukturänderung im modernen Bergbau erhöht das Notfallpotenzial ohne Verfügbarkeit einer dem öffentlichen Rettungsdienst vergleichbaren Notfallrettung unter Tage, bei zusätzlich deutlich verlängerten Rettungszeiten.

**Fragestellung:**

Kann die Grubenwehr zur medizinischen Notfallrettung unter Tage ertüchtigt werden?

**Material und Methoden:**

Ein auf typische Notfälle optimiertes medizinisch-taktisches Rettungsschema nebst Ausrüstung wurde entwickelt und medizindidaktisch optimiert in 16 Unterrichtseinheiten geschult. Objective Structured Practical Examinations (OSPE) von 3 geschulten Grubenwehrtrupps a 4 Wehrleuten wurden mittels identischer Prüfung von zufällig ausgewähltem Rettungsdienstreferenzpersonal (17 Teilnehmer unterschiedlicher Ausbildungsniveaus) verglichen.

**Ergebnisse:**

Das medizinisch-taktische Rettungsschema beinhaltet Vitalfunktions- und Bodycheck, Reanimation mit Defibrillation, nasale und intraossäre Medikamentengabe, Atemwegssicherung, Thoraxpunktion, Blutstillung, Tourniquet, Reposition, Schienung sowie Transportlagerung mit Wärmeerhalt. In der OSPE-Prüfung erzielte die Grubenwehr (Mittelwert [M] = 3,42, 95 %-Konfidenzintervall [KI_95_ _%_] = [3,24; 3,60]) gleiche Ergebnisse wie der Rettungsdienst höheren Ausbildungsniveaus (M = 3,28, KI_95_ _%_ = [3,09; 3,46]), jedoch deutlich bessere Ergebnisse als Rettungssanitäter (M = 2,43, KI_95_ _%_ = [2,10; 2,77]). Das Kompetenzniveau der Grubenwehr blieb nach einem 6‑monatigen übungsfreien Intervall stabil (M = 3,54, KI_95_ _%_ = [3,31; 3,73]).

**Diskussion:**

Das erzielte Kompetenzniveau der ausgebildeten Wehrleute nach taktischem Minenrettungskurs ist mit dem öffentlichen Rettungsdienst innerhalb des eng definierten Behandlungsschemas vergleichbar. Die Grubenwehr kann unter Anwendung medizinischer Notkompetenz ein geeignetes Instrument sein, um die Lücke der professionellen Notfallrettung unter Tage zu schließen.

**Zusatzmaterial online:**

Die Onlineversion dieses Beitrags (10.1007/s00063-021-00861-w) enthält die Tabellen A1–A3.

## Abstract (en)

**Background:**

Structural changes in modern mining industry increase the potential for medical emergencies. Furthermore, rescue times in mining are prolonged and public medical resources are not consistently available.

**Objectives:**

We sought to train mine rescue brigade lay people to cover medical emergencies in mining.

**Materials and methods:**

A standardized tactical–medical approach including specific equipment was developed and taught in a didactically optimized way in 16 lessons. Objective Structured Practical Examinations (OSPE) were conducted in 3 groups of 4 mine rescue personnel and compared to the identical exam of the reference group (17 paramedics of different educational and experience levels).

**Results:**

The tactical–medical scheme includes vital functions and body checks, advanced life support, nasal and intraosseous drug administration, airway management, thoracocentesis, bleeding control, tourniquet, fracture repositioning, splinting and transport bedding. In OSPE evaluation, the scores of the trained mine rescue personnel (mean [M] = 3.42, 95% confidence interval [CI_95%_] = [3.24; 3.60]) were statistically equal to those of advanced paramedics (M = 3.28, CI_95%_ = [3.09; 3.46]), but much better than basic German paramedic level (M = 2.43, CI_95%_ = [2.10; 2.77]). Competency retention of mine rescue personnel remained on the same level after a period of 6 months without further training (M = 3.54, CI_95%_ = [3.31; 3.73]).

**Conclusion:**

The competency level after the Tactical Mining Rescue Course is comparable to the advanced paramedics level in the spectrum of competencies addressed. Medical lay people can be trained to deliver an acceptable treatment level within a clearly defined treatment algorithm, and thus potentially close the gap towards professional medical emergency rescue in mining.

## Hintergrund

Mit dem Wandel des deutschen Bergbaus von ehemals wenigen großen Förderbetrieben hin zu kleinen Betrieben mit breitem Spektrum (neuartige Rohstoffe, Forschung, Rekultivierung, Deponierung, Endlagerung) kommt es auch zwangsläufig zu Strukturänderungen im Grubenrettungswesen. Bei derzeit rund 500 jährlichen Unfällen mit mehr als 3 Tagen Arbeitsunfähigkeit [2] stellt sich die Frage flächendeckender medizinischer Erstversorgungsmöglichkeiten unter Tage bei zumeist fehlender betrieblicher Vorhaltung. Aufgrund von Gefährdungsanalysen aus vergangenen Einsätzen von Grubenwehren ist bekannt, dass die medizinische Laienrettung bis zur Übergabe des Patienten an einen Notarzt regelhaft weit mehr als die gesetzliche Hilfsfrist von 12 min [20] beträgt. Bei der Betrachtung des Einsatzspektrums bestehen zudem deutliche Unterschiede zwischen zivilem Bereich und dem Bergbau mit überwiegend traumatologischen Notfällen [18, 22]. Das derzeit geforderte Qualifikationsniveau gemäß der Leitlinie für das deutsche Grubenrettungswesen (betrieblicher Ersthelfer) ist daher bei den Einsatzkräften der Grubenwehr aufgrund der zu erwartenden Verletzungsschwere nicht mehr ausreichend [12]. Darüber hinaus ist der Einsatz öffentlicher Notfallrettung im Bergbau nicht gewährleistet: Feuerwehren fehlen hauptsächlich Langzeitatemschutztechnik und Kommunikationsmittel. Dem Rettungsdienst erschweren neben der Ausrüstungsproblematik die Regelungen der Berufsverbände den Einsatz in Gefahrenbereichen. Ein Einsatz unter Tage ist somit nur sehr beschränkt und auf freiwilliger Basis im Einzelfall möglich. Daher braucht es im Zuge der Transformation des modernen Bergbaus in Europa neue Konzepte, um eine adäquate Sicherstellung der Notfallrettung zu gewährleisten.

Als Grundlage für die Konzeptentwicklung wurden durch ähnliche Rahmenbedingungen in Hochrisikobereichen mit begrenzten Ressourcen an Material und Personal, komplexen Traumata, langen Transportwegen sowie fehlender Erstsichtung durch einen Arzt Ansätze der taktischen Medizin aus dem militärischen (Tactical Combat Casualty Care, TCCC; [6]) bzw. polizeilichen (Tactical Emergency Medical Support, TEMS; [3, 6, 10, 15]) Bereich verfolgt. Aber auch hier konnten nur grundlegende Vorgehensweisen berücksichtigt werden, da beim Einsatz der Grubenwehr abweichende Herausforderungen wie Enge, Dunkelheit, Nässe und Schmutz, Senkrechtrettung und nichtatembare Atmosphäre viel mehr als die taktische Lage im Vordergrund stehen. Daher wurden bestehende Modelle aus zivilen Bereichen mit Elementen der taktischen Medizin evaluiert und in die Entwicklung des Rettungsschemas und des Kurskonzepts einbezogen. Besonders seien hier die Information Erste Hilfe in Offshore-Windparks der Deutschen Gesetzlichen Unfallversicherung [23] sowie die Konzepte der taktischen Alpinmedizin des Österreichischen Bergrettungsdiensts [1, 15] genannt. Auch wurden die Ausbildungsrichtlinien des Sanitätsdienstes der Bundeswehr mit einem vergleichbaren Qualifizierungsniveau bei der Erarbeitung berücksichtigt [7, 10, 11]. Dennoch mussten Einsatzkonzept, Behandlungsspektrum, Ausrüstung und Ausbildungscurriculum für die Besonderheiten unter Tage vollständig neu entwickelt werden.

### Fragestellung

Können Mitglieder der Grubenwehr ohne vorbestehende notfallmedizinische Ausbildung mit einem 2‑tägigen standardisierten Curriculum ein praktisches Kompetenzniveau und -spektrum zur Notfallrettung unter Tage erreichen, dass mit dem reellen präklinischen Patientenversorgungsstandard des Rettungsdiensts vergleichbar ist?

## Studiendesign und Untersuchungsmethoden

Zur Definition des notwendigen Behandlungsspektrums wurde das Einsatzspektrum der Grubenwehren in den letzten Jahren herangezogen, die neben der Brandbekämpfung und technischen Hilfeleistung auch regelmäßig für die Notfallrettung eingesetzt wurden. Tabelle 1 zeigt dabei exemplarisch Verletzungsarten und Einsatzzeiten bei Einsätzen der Grubenwehren in Sachsen und Sachsen-Anhalt in den Jahren 2018–2019.

**Table 1:** 

Datum	Bergwerk	Unfallursache	Verletzung	Einsatzdauer(Unfallzeitpunkt bis zur Übergabe an den Rettungsdienst über Tage)
03/2019	1	Absturz	Polytrauma	11 h
04/2019	2	Suizid	Strangulation	2,5 h
11/2019	3	Verpuffung	Verbrennung, Inhalationstrauma	1 h
06/2018	4	Unfall mit Transportmittel	Polytrauma	1,5 h
07/2018	5	Einklemmung	Polytrauma	3 h
08/2018	4	Brand	Rauchgasvergiftung	1,5 h

Auch im österreichischen Bergbau ist die aktuelle Entwicklung vergleichbar, dort musste in den letzten 5 Jahren bereits eine Zunahme von Unfällen registriert werden [5]. Von rund 150 Unfällen pro Jahr sind knapp 30 % schwer und knapp 1 % mit tödlichem Ausgang. Typische Unfallursachen sind zurückzuführen auf Arbeitsmittel und -stoffe, Förderung und Transport, Steinfall, elektrischen Strom und Sprengarbeiten. Auftretende Verletzungen sind dabei zum überwiegenden Teil Hand- und Fußverletzungen (rund 45 %) gefolgt von Kopf- und Körperstammverletzungen.

Um dieses Verletzungsmuster und potenzielle Erkrankungen einer Erstbehandlung zuzuführen, deren primäre Ziele die Verhinderung weiterer Verschlechterung, die Stabilisierung der Vitalfunktionen und die effiziente Verletztenbergung sind, wurden folgende Prioritäten zur Entwicklung eines komprimierten Handlungsalgorithmus festgelegt:

1. Einschätzung akuter vitaler Gefährdung;
2. Blutstillung;
3. kardiorespiratorische Stabilisierung;
4. Schmerztherapie;
5. raumluftunabhängige Atemunterstützung und ggf. Beatmung;
6. Wärmeerhalt;
7. Transportlagerung mit fixiertem Equipment;
8. Möglichkeit der Schleif- und Senkrechtrettung.

Neben der Entwicklung des Handlungsalgorithmus musste auf das Einsatzspektrum und die genannten Besonderheiten abgestimmtes Material und Ausrüstung zusammengestellt und in den Algorithmus integriert werden. Folgende Anforderungen wurden dabei priorisiert:

1. geringe Größe und Gewicht;
2. Resistenz gegenüber Feuchtigkeit, Schmutz und mechanischer Belastung;
3. Bedienbarkeit unter eingeschränkten Sichtverhältnissen;
4. Integrationsmöglichkeit in die Transportanforderungen;
5. Bedienbarkeit während des Transports.

Weiterhin musste nach Entwicklung von Behandlungsalgorithmus und abgestimmter Ausrüstung ein medizindidaktisch optimiertes Ausbildungskonzept [16] erstellt werden, das folgende Anforderungen erfüllen musste:

1. standardisierter identischer Trainingsablauf;
2. klar abgrenzbare didaktische Einheiten zur sequenziellen Vermittlung praktischer Fertigkeiten;
3. für den medizinischen Laien transparent vermittelbare und mess-/prüfbare Lernziele;
4. Entwicklung und Schulung einer logischen medizinischen Kausalkette unter Vermeidung von Überfrachtung mit medizinischem Hintergrundwissen.

Nach Probandenaufklärung und positiver ethischer wie juristischer Stellungnahme der TU Bergakademie Freiberg wurden 3 Trupps der Grubenwehr mit je 4 Wehrleuten unterschiedlicher Bergbaubetriebe rekrutiert. Diese absolvierten das entwickelte und im Ergebnisteil dargestellte Ausbildungscurriculum. Direkt danach wurden die Teilnehmer einer standardisierten Objective Structured Practical Examination (OSPE) am Modell und am Schauspielpatienten unterzogen. Um das OSPE-Ergebnis in Bezug auf den tatsächlichen Patientenversorgungsstandard im Rettungsdienst einzuordnen, wurde zufällig ausgewähltes Rettungsdienstpersonal unterschiedlicher Erfahrungs- und Ausbildungsniveaus (Rettungssanitäter, Rettungsassistenten und Notfallsanitäter) als Referenzgruppe der identischen OSPE-Prüfung unterzogen. Die einzelnen OSPE-Stationen wurden von verschiedenen Prüfern abgenommen, jede einzelne OSPE-Station jedoch für Grubenwehrteilnehmer und Rettungsdienstreferenzgruppe vom identischen Prüfer. Die Prüfungschecklisten enthielten dabei mehrere Items für jede praktische Fertigkeit, die Bewertung der Items erfolgte mit einer 4‑Punkte-Skala (schlecht, befriedigend, gut, sehr gut). Null Punkte wurden vergeben, wenn ein Teilaspekt nicht durchgeführt wurde.

Die statistische Auswertung und die graphische Darstellung erfolgte mittels R v4.0.3 [19]. Die jeweiligen Gesamtscores der 7 OSPE-Stationen wurden aus den Mittelwerten der Einzelnote für jedes Prüfungsitem mit gleicher Gewichtung ermittelt. Verglichen wurden die Gesamtscores der OSPE-Stationen 1–6 mittels einer Varianzanalyse mit Messwiederholung, wobei die Teilnehmer- bzw. Referenzgruppe und die jeweilige OSPE-Station als feste Faktoren definiert wurden [24]. Post hoc wurde der Tukey-Test verwendet [9]. Die Station Nr. 7 als Lagerungsstation in der Schleiftrage kommt im Übertagerettungsdienst nicht vor und wird daher vom Vergleich exkludiert. Unterschiedliches Material der Prüfungsgruppen Grubenwehr und Rettungsdienst wurde für den OSPE soweit modifiziert, dass in jeder Gruppe nur bekanntes Material verwendet wurde (z. B. Austausch des sternalen intraossären Zugangssets der Grubenwehr gegen die Rettungsdienststandardausrüstung Intraossär-Bohrmaschine). Schließlich erfolgte eine Subgruppenanalyse nach Ausbildungs- und Erfahrungsstand des Rettungsdiensts mit der beschriebenen Methodik.

## Ergebnisse

**Figure 1:**
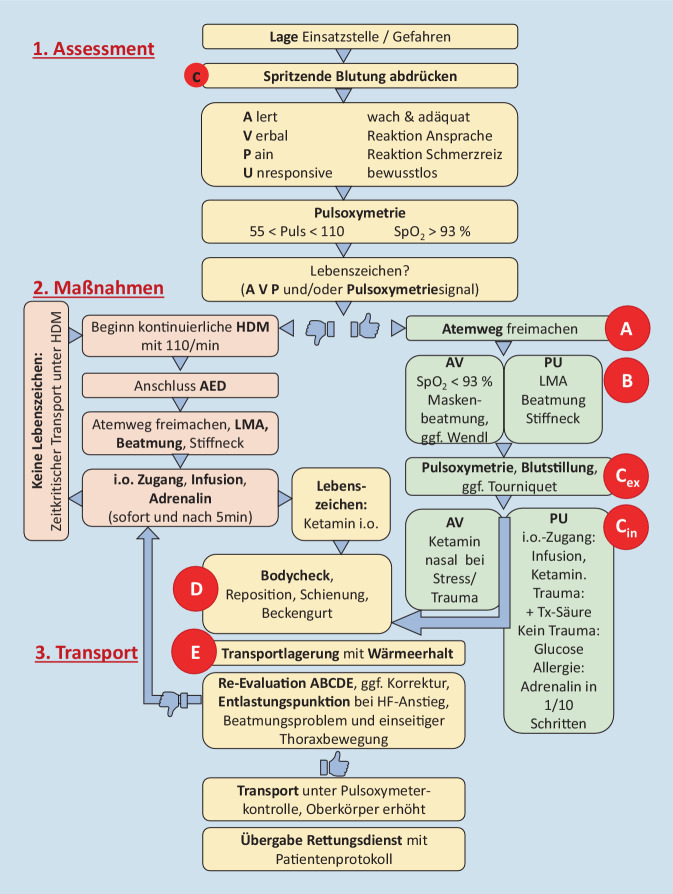

Das für das Einsatzspektrum unter Tage entwickelte standardisierte medizinisch-taktische Rettungsschema ist in Abb. 1 dargestellt, das prinzipielle Vorgehen nach cABCDE-Algorithmus wurde dabei modifiziert.

**Figure 2:**
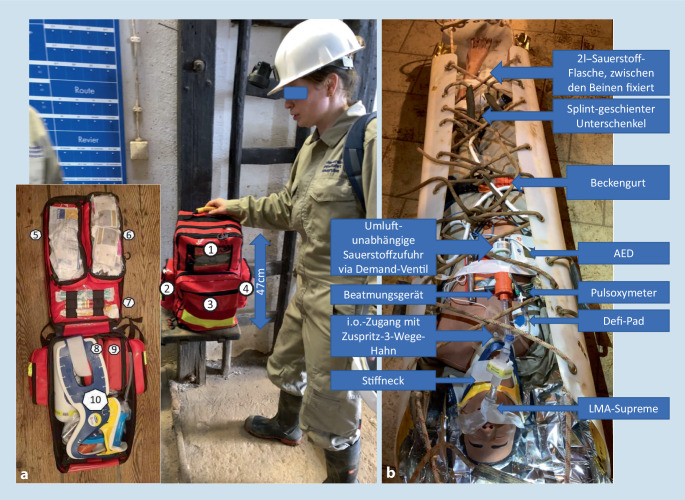

Die darauf abgestimmte Ausrüstung anhand der im Methodenteil aufgestellten Anforderungen ist in Abb. 2 dargestellt.

Die gesamte Ausrüstung bis auf die Roll-Schleif-Trage ist in dem abgebildeten Rucksack prozessorientiert verstaut und hat die Außenmaße von 47 × 36 × 26 cm und ein Gesamtgewicht von 12,5 kg. Die Roll-Schleif-Trage der Firma Kendler hat eine Länge von 2,4 m und ein Packmaß von ca. Ø 0,3 × 1,0 m. In der Rolltrage wurde eine Rettungsfolie für den passiven Wärmeerhalt eingeklebt und in Verbindung mit einem Aktivwärmepad eine modifizierte Hibler-Wärmepackung in das Rettungssystem integriert.

Unter Maximierung von Anwendersicherheit und Reduktion von Ausbildungsaufwand wurde z. B. auf einen übungsintensiven intravenösen Zugang verzichtet und stattdessen ein sternaler Intraossärzugang (EZ-IO T.A.L.O.N.) verwendet. Neben der einfachen Anwendung bestehen Vorteile der Sicherung und Erreichbarkeit während des Transports. Eine vollständig entlüftete Kolloidinfusion wurde ohne störende Schwerkraftinstallation unter dem Gesäß platziert und durch das Patienteneigengewicht autoinfundiert. Zur Beatmung bzw. umluftunabhängigen druckunterstützten Spontanatmung wurde ein OXYLATOR FR 300B mit dem dazugehörigen Demandventil (CPR Medical Inc., Canada) eingesetzt. Patientenseitig wurde nichtinvasiv mit einer stabilen blockbaren Gummimaske gearbeitet, invasiv mit einer Larynxmaske (LMA) Supreme (Größen 4 und 5), da diese im Gegensatz zum Larynxtubus einen robusteren Cuff und überlegene Platzierungs- und Abdichteigenschaften aufweist [21]. Für den Transport wurde alle relevante Medizintechnik (Defibrillator, Pulsoxymeter, Beatmungsgerät und Zuspritzport des i.o.-Zugangs) im Sternumbereich mit Pflaster fixiert, sodass auch unterwegs die Bedienbarkeit und der Überblick über die Vitalfunktionen jederzeit und an nur einer Stelle gewährleistet ist. Zur Fixierung aller Maßnahmen stand aus Gründen der Nässe- und Schmutzresistenz lediglich 5 cm breites Silk-Rollenpflaster zur Verfügung. Die Auswahl der Medikamente beschränkte sich auf Volumenersatz mittels 4 %iger Gelatinelösung (2-mal 500 ml), Tranexamsäure (2-mal 500 mg), Ketamin S (3-mal 50 mg), Adrenalin (3-mal 1 mg) und Glukose 40 % (3-mal 10 ml). Dabei wurden zur Vermeidung von Fehlern durch Stress und begrenztes Fachwissen die Wirkstoffeinheiten so gewählt, dass für den erwachsenen Patienten stets eine ganze Ampulle aufgezogen und verabreicht wird.

**Figure 3:**
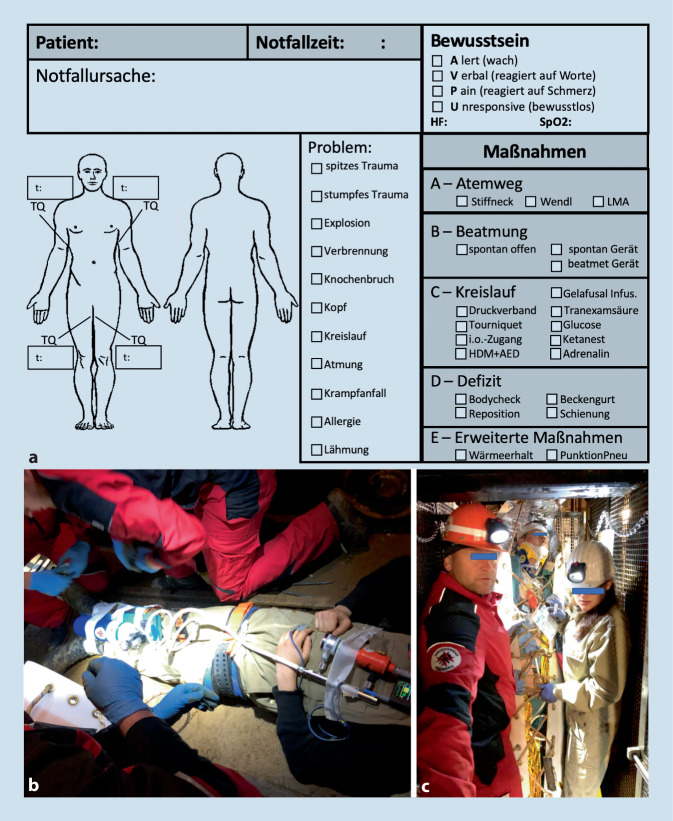

Zur Dokumentation der Maßnahmen wurde eine laminierte Patientenanhängekarte im Format A5 entwickelt (Abb. 3).

Das zur Schulung des Rettungsschemas und Anwendung der abgestimmten Ausrüstung entwickelte medizindidaktisch optimierte Ausbildungscurriculum wurde in 7 Kompetenzbereiche untergliedert, die mit modifizierter Peyton-Methode [16] sequenziell vermittelt wurden (Tab. 2). Training und Szenarien fanden vollständig unter Tage in realistischen Bedingungen statt.

**Table 2:** 

Einzelne Kompetenzbereiche	Inhalt
Initiales Assessment	c‑AVPU-ABCDE-Prozesskette, Pulsoxymetrie, Blutung komprimieren
BLS mit AED	Herzdruckmassage und Algorithmus, AED-Anwendung ohne Beatmung
A, B	Beatmung mit Maske/Kopfgurt und Beatmungsgerät, Wendl, Stiffneck, LMA-Insertion
C‑out	Bodycheck, Blutstillung, Tourniquet
C‑in	MAD, i.o.-Zugang, Infusion, Medikamente (Tranexamsäure, Adrenalin, Ketanest S, Glukose)
D	Reposition, Schienung, Beckengurt, Entlastungspunktion
E	ABCDE-Reevaluation, Transportverpackung mit Wärmeerhalt

### Vergleich der Prüfungsergebnisse der praktischen Skills zwischen Interventionsgruppe Grubenwehr und Referenzgruppe Rettungsdienst

**Figure 4:**
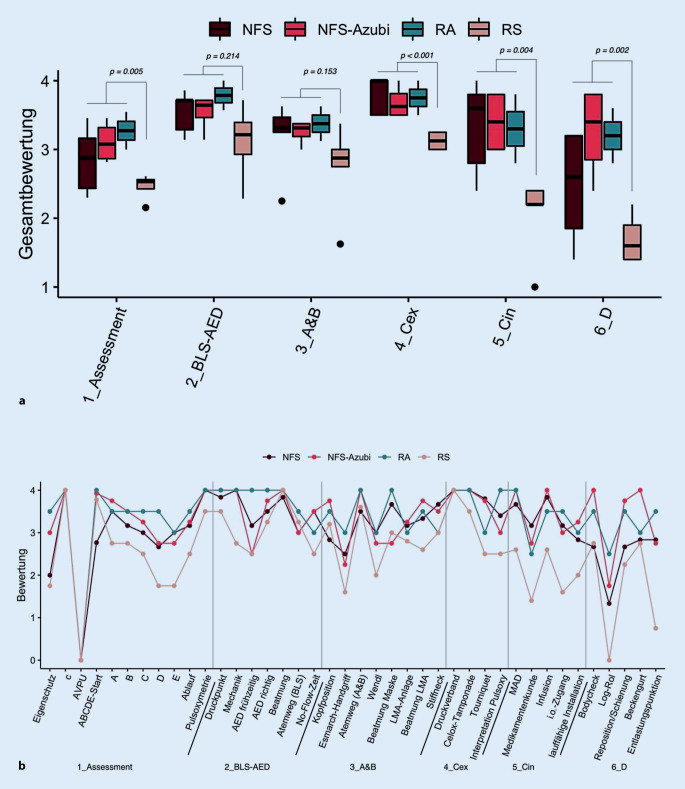

Im Vergleich zeigten sich deutliche Subgruppenunterschiede innerhalb der Referenzgruppe Rettungsdienst und die Referenzteilnehmer mit dem Qualifizierungsniveau Rettungssanitäter schnitten schlechter ab (*p* < 0,001) als die restlichen Teilnehmergruppen mit den Qualifizierungsniveaus Notfallsanitäter, Notfallsanitäter in Ausbildung und Rettungsassistenten, speziell in den Stationen 1, 4, 5 und 6 (Abb. 4). Zwischen Notfallsanitätern, Rettungsassistenten und in Ausbildung befindlichen Notfallsanitätern konnte hingegen kein Unterschied festgestellt werden (*p* > 0,05; Abb. 4).

**Figure 5:**
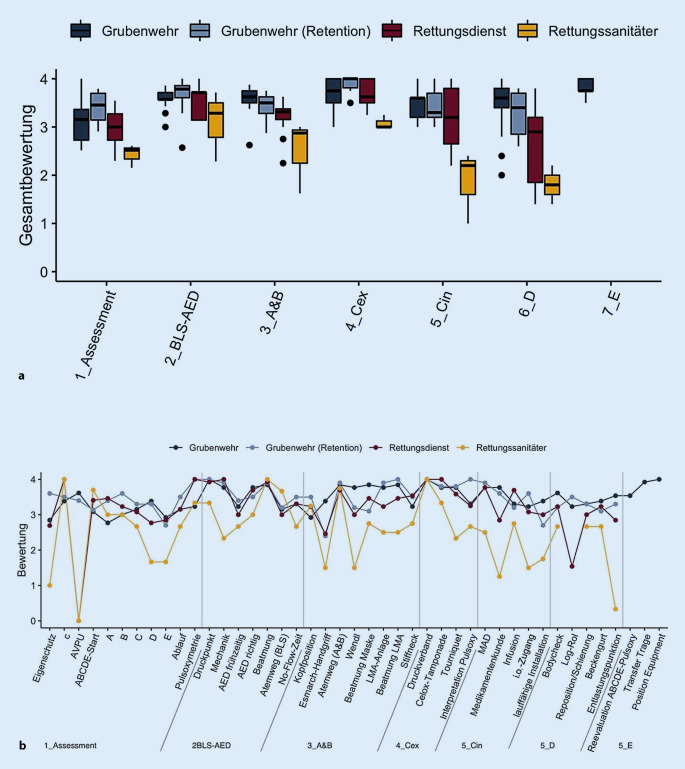

Für die weitere Auswertung wurde daher eine Gesamtreferenzgruppe Rettungsdienst aus Notfallsanitätern, Notfallsanitätern in Ausbildung und Rettungsassistenten gebildet, die Rettungssanitäter hingegen wurden als separate Gruppe betrachtet. Im Vergleich aller Testitems der OSPE-Stationen 1–6 erzielte die Interventionsgruppe Grubenwehr (M = 3,42; KI_95_ _%_ = [3,24; 3,60]) ein gleiches Ergebnis wie die Gruppe Rettungsdienst (M = 3,28; KI_95_ _%_ = [3,09; 3,46]), jedoch ein signifikant besseres als die Rettungssanitäter (M = 2,43; KI_95_ _%_ = [2,10; 2,77]). In der Einzelbetrachtung der OSPE-Stationen (Tab. 2) unterschieden sich die beiden Gruppen Grubenwehr und Rettungssanitäter in den Stationen 3, 4, 5 und 6, und Grubenwehr und Rettungsdienst lediglich in Station 6 (Abb. 5; Tab. A1 bis A3 im Onlinezusatzmaterial). Nach einem 6‑monatigen übungsfreien Intervall wurden die geschulten Grubenwehrmitglieder erneut der identischen OSPE-Prüfung unterzogen. Die dabei erzielten Ergebnisse (M = 3,54; KI_95_ _%_ = [3,31; 3,73]) zeigten weder einen Unterschied zu den Ergebnissen der Grubenwehr direkt nach dem Training noch zu der Referenzgruppe Rettungsdienst.

## Diskussion

Bei der Erstellung des vorliegenden Ausbildungskonzepts wurden Vorgehensweisen aus dem militärischen sowie zivilen Bereich übernommen und an die Bedürfnisse und Anforderungen der Grubenwehr für die Verhältnisse unter Tage angepasst. Wir konnten zeigen, dass es möglich ist, mit einer einmaligen standardisierten, medizindidaktisch optimierten praktischen Ausbildung über knapp 2 Tage ein Kompetenzniveau zu erzielen, das in Bezug auf die im engen Rahmen vermittelten Kompetenzen dem Patientenversorgungsstandard im öffentlichen Rettungsdienst gleichwertig ist. Retentionsmessungen mittels Nachprüfungen nach 6 Monaten ohne zwischenzeitliches Training zeigten ein stabiles Kompetenzniveau. Unser Ausbildungskonzept zur Qualifizierung medizinischer Laien in der Untertagerettung besteht in der Vermittlung eines relativ einfachen Handlungsalgorithmus unter Verbindung des AVPU- und c(x)ABCDE-Schemas [14]. Innerhalb dieses Schemas werden hinsichtlich Komplexitätsreduktion speziell entwickelte praktische Fertigkeiten aneinandergereiht. Vergleichbare Untersuchungen zur Effektquantifizierung der einmaligen Vermittlung wenig komplexer medizinisch-praktischer Fertigkeiten unter Anwendung eines medizindidaktisch optimierten Curriculums erzielen ähnliche Ergebnisse [8], wobei komplexe akutmedizinische Fertigkeiten einen deutlich höheren Übungsaufwand erfordern [13]. In den Abschlussszenarien der entwickelten Trainings wurden mithilfe von Schauspielpatienten und Rettungsdienst sehr realitätsnahe Simulationen durchgeführt, die von manchen Mitwirkenden nicht als Übung erkannt wurden. Limitierend ist zu konstatieren, dass die klinische Performanz im Zusammenspiel der erlernten praktischen Fertigkeiten nur qualitativ im Rahmen des Abschlussszenarios geprüft wurde. Dabei waren alle Grubenwehrtrupps in der Lage, ihre praktischen Kompetenzen im Rahmen des vorgegebenen taktisch-medizinischen Rettungsschemas selbstständig, flüssig und korrekt anzuwenden, sodass der standardisierte Patient innerhalb von 15 min komplikationsfrei versorgt und sich für die Übergabe an den Rettungsdienst auf dem Transport befand. Der Sauerstoffvorrat (2 l; 200 bar; 3,5 kg Stahl) war dabei gerade so ausreichend und könnte durch eine 300 bar-Composite-Flasche optimiert werden. Quantitativ konnte lediglich das Kompetenzniveau der einzelnen praktischen Fertigkeiten geprüft werden. Dies war bei der Prüfung der Referenzgruppe Rettungsdienst aber auch der Fall. Daher stehen Erfahrungen mit der Anwendung der erlernten Kursinhalte in der klinischen Realität noch aus. Jedoch soll die medizinisch-taktische Rettung durch die Grubenwehr nur als Notkompetenz bis zur Übergabe an den örtlichen Rettungsdienst und in den engen Schranken des vermittelten Behandlungsalgorithmus erfolgen. Nach den bisherigen Ergebnissen dürfte im Vergleich zur Alternative – lediglich Bergung ohne medizinische Intervention – das Risiko eines schlechten Patientenoutcomes nach Anwendung der erworbenen medizinischen Kompetenzen durch Wehrleute der Grubenwehr deutlich minimiert sein.

### Zulassungsverfahren

Die Unternehmerpflicht zur Gewährleistung einer medizinischen Notversorgung ergibt sich aus § 11 Abs. 1 Nrn. 4 bis 7 ABBergV [17]. Art und Umfang der Maßnahmen sind im Rahmen der betriebsbezogenen Gefährdungsanalyse festzulegen und in den betrieblichen Notfallplan nach § 11 Abs. 1 Nr. 6 ABBergV [17] aufzunehmen. Die Zulassung der geplanten Maßnahmen erfolgt durch die zuständige Genehmigungsbehörde, im Freistaat Sachsen z. B. durch das Sächsische Oberbergamt. Dies kann in Form eines Sonderbetriebsplans (SBP) erfolgen, der durch den jeweiligen Unternehmer oder betriebsübergreifend bei gemeinschaftlicher Grubenwehr eingereicht wird. Besteht kein eigenständiger SBP, ist die betriebliche Regelung zur medizinischen Ersthilfe Gegenstand des Hauptbetriebsplans nach § 55 Abs. 1 Nr. 3 BBergG [4]. Inhalt ist eine genaue Beschreibung der Einsatzmöglichkeiten der Grubenwehr, wozu auch die persönliche Qualifikation für Ersthilfemaßnahmen gehört. Zur Beurteilung dieser Voraussetzungen kann hierbei auf ein standardisiertes Ausbildungs- und Trainingskonzept für genau definierte Maßnahmen der medizinischen Erstversorgung verwiesen werden, wie es in diesem Beitrag entwickelt wurde.

Die erweiterte Einsatzmöglichkeit der Grubenwehr für Notfallmaßnahmen, die ansonsten Notfallsanitätern und Ärzten vorbehalten sind, bleibt allerdings in Bezug auf letztere nachrangig, da sie nur in einer Notstandssituation gerechtfertigt werden kann. Im Einsatzfall muss dabei immer abgewogen werden, ob die indizierten Maßnahmen durch einen Notarzt geleistet werden können, ob über geeignete Kommunikationswege eine Konsultation oder Anleitung durch einen Arzt erfolgen kann oder ob zur Abwendung einer unmittelbaren Gefahr für Leben und Gesundheit eines Verunfallten die dafür qualifizierte Grubenwehr im Rahmen der Notkompetenz handeln muss.

## Schlussfolgerung

Als Grundlage zur Einreichung des Sonderbetriebsplans und als Vorlage für Unfallversicherungsträger kann künftig dieses Ausbildungskonzept dienen, um die Lücke bisher nicht vorhandener erweiterter Erster Hilfe im Bergbau zu schließen und einen aktuell validierten und standardisierten Stand der allgemein anerkannten Regeln der medizinischen Hilfe, Ausbildung und Rettungstechnik im Bergbau zu definieren. Es erfüllt zudem grundlegende Anforderungen für vergleichbare Einsatzumgebungen mit erschwertem Personal- und Materialzugang wie z. B. in Forstwirtschaft, Bergrettung, Leitungs- und Windenergieanlagenbau.

## Supplementary Information




