# Supplementary material for: Development and validation of a course concept for Tactical Medical Mining Rescue: Standardized training curriculum for mine rescue teams
Source: Med Klin Intensivmed Notfmed. 2021 Sep 20;117(7):531–41. [Article in German] doi: 10.1007/s00063-021-00861-w (PMC9553787; doi:10.1007/s00063-021-00861-w)
Supplement: Supplementary file 1 [file 63_2021_861_MOESM1_ESM.pdf]

Zusatzmaterial

Tabelle A1: Vergleich der Gesamtbewertung nach OSPE-Station

	**Grubenwehr**			**Rettungs-dienst**			**Vergleich**	
	n	***M***	***SD***	***n***	***M***	***SD***	***t***	***p (holm)***
1_Assessment	13	3.14	0.44	16	2.87	0.44	1.67	0.318
2_BLS-AED	13	3.58	0.24	16	3.46	0.42	0.95	0.658
3_A&B	13	3.58	0.32	17	3.10	0.51	3.17	0.023
4_Cex	13	3.71	0.32	15	3.58	0.36	0.99	0.658
5_Cin	13	3.49	0.33	17	2.97	0.83	2.39	0.102
6_D	13	3.42	0.64	16	2.56	0.87	3.05	0.025
7_E	13	3.79	0.20					

Tabelle A2: Vergleich der Rettungsdienstgruppen

	NFS			NFS-Azubi			RA			RS		
	n	*M*	*SD*	*n*	*M*	*SD*	*n*	*M*	*SD*	*n*	*M*	*SD*
1_Assessment	6	2.84	0.47	4	3.11	0.31	2	3.27	0.39	4	2.46	0.21
2_BLS-AED	6	3.55	0.32	4	3.54	0.27	2	3.79	0.30	4	3.11	0.60
3_A&B	6	3.21	0.49	4	3.25	0.18	2	3.38	0.35	5	2.73	0.66
4_Cex	5	3.80	0.27	4	3.69	0.24	2	3.75	0.35	4	3.13	0.14
5_Cin	6	3.33	0.68	4	3.40	0.46	2	3.30	0.71	5	2.04	0.59
6_D	6	2.47	0.83	4	3.25	0.68	2	3.20	0.57	4	1.70	0.38

NFS Notfallsanitäter, ***RA*** Rettungsassistent, ***RS*** Rettungssanitäter

Tabelle A3: Vergleich der Einzel-Items der OSPE-Prüfung zwischen der Gruppe Grubenwehr (nach dem Training) und Rettungsdienst (ohne Rettungssanitäter).

		Grubenwehr			Rettungsdienst		
OSPE Hauptgruppen		n	M	SD	n	M	SD
1_Assessment	Eigenschutz	13	2.846	1.281	16	2.375	1.746
	c	13	3.385	1.044	16	4	0
	AVPU	13	3.615	0.65	16	0	0
	ABCDE-Start	13	3.085	0.948	16	3.462	0.913
	A	13	2.769	0.927	16	3.375	0.619
	B	13	3	1	16	3.188	0.75
	C	13	3.154	0.801	16	3	0.73
	D	13	3.385	0.87	16	2.562	0.814
	E	13	2.923	0.954	16	2.625	0.806
	Ablauf	13	3.154	0.801	16	3.062	0.772
	Pulsoxymetrie	13	3.231	1.166	16	3.875	0.5
2_BLS-AED	Druckpunkt	13	4	0	16	3.812	0.544
	Mechanik	13	3.769	0.439	16	3.688	0.873
	AED frühzeitig	13	3.231	0.832	16	2.938	0.854
	AED richtig	13	3.769	0.599	16	3.562	1.031
	Beatmung	13	3.846	0.376	16	3.938	0.25
	Atemwegs-management (BLS)	13	3.154	0.801	16	3.125	0.719
	No-Flow-Zeit	13	3.308	0.751	16	3.188	0.834
3_A&B	Kopfposition	13	2.923	0.641	17	3.235	0.903
	Esmarch-Handgriff	13	3.385	0.65	17	2.235	1.348
	Atemweg (A&B)	13	3.846	0.555	17	3.706	0.772
	Wendl	13	3.769	0.439	17	2.647	1.057

		Grubenwehr			Rettungsdienst		
	Beatmung Maske	13	3.846	0.376	17	3.294	0.92
	LMA-Anlage	13	3.769	0.439	17	3.059	0.659
	Beatmung LMA	13	3.846	0.376	17	3.235	0.831
	Stiffneck	13	3.231	0.832	17	3.353	0.702
4_Cex	Druckverband	13	4	0	15	4	0
	Celox-Tamponade	13	3.769	0.439	15	3.867	0.352
	Tourniquet	13	3.769	0.439	15	3.333	0.816
	Interpretation Pulsoxy	13	3.308	1.182	15	3.133	0.834
5_Cin	MAD	13	3.769	0.599	17	3.471	0.874
	Medikamenten-kunde	13	3.769	0.439	17	2.471	1.125
	Infusion	13	3.308	0.751	17	3.471	0.8
	io-Zugang	13	3.231	0.599	17	2.706	1.263
	lauffähige Installation	13	3.385	0.65	17	2.706	0.985
6_D	Bodycheck	13	3.615	0.65	16	3.125	0.719
	Log-Rol	13	3.231	0.927	16	1.25	1.571
	Reposition /Schienung	13	3.308	0.855	16	2.938	0.929
	Beckengurt	13	3.385	0.768	16	3.125	1.025
	Entlastungspunktion	13	3.538	0.877	16	2.375	1.544
7_E	Reevaluation ABCDE-Pulsoxy	13	3.538	0.519	17	0	0
	Transfer Trage	13	3.923	0.277	17	0	0
	Position Equipment	13	4	0	17	0	0
